# Wound of the Heart

**Published:** 1828-12

**Authors:** 


### WOUND OF THE HEART.

### *Wound of the Heart, in which the Patient survived the Accident ten days.*

Victor Janson, sixteen years of age, on the 8th of September wounded himself accidentally in pulling a knife from one of his companions; but, as he felt no pain, he supposed he had only cut his waistcoat. He laid the knife upon the table, walked out into the court, and remained there ten minutes without even thinking about the accident. At the end of this time he observed his clothes stained with blood; he vomited, and fell to the ground.

He was conveyed to La Charité. The blood which flowed at this time was florid. When brought to the hospital, his face was pale, his lips colourless, eyelids drooping, respiration short and frequent; pulse small, frequent, and compressible; the shirt and clothes of the patient bathed in blood Between the fourth and fifth ribs of the left side, near two fingers' breadth from the sternum, was a transverse aperture, from six to seven lines in length. It was remarked that the wound of the integuments did not correspond exactly with that of the muscles or pleura; and this want of parallelism prevented the blood from having a free exit. In order to determine the exact direction in which the instrument had penetrated, and the hemorrhage continuing, the extremity of a covered director was introduced into the wound; and seeing that it tended to run from within outwards, and from above downwards, it was withdrawn, without introducing it within the chest. The lips of the wound were gently approximated by means of adhesive straps, and a compress and bandage applied, moderately tight. The left and back part of the chest emitted a dull sound on percussion; the respiratory murmur was feeble at the upper part, and entirely wanting beneath. On the right the sound was clear, and the respiratory murmur very perceptible. The patient being laid on his back, the sound was sufficiently clear above and in front, but dull beneath and at the side, where the respiratory murmur was lost. These different signs clearly pointed out considerable extravasation of blood into the chest. The patient continued all this time with his eyes shut, and insensible. He was bled, and sinapisms were applied to the calves of the legs, the feet rolled in warm cloths, and the hands retained some minutes in warm water.

Next morning it was found that he had slept two hours during the night. Some reaction had manifested itself. He was directed by M. Boyer to be bled three times, viz. immediately, at mid-day, and at night. The first produced a momentary relief; the second and third also somewhat diminished the oppression.

The symptoms still remaining, on the third day he was again bled, and the venesection once more repeated at night.

On the 11th (the fourth day), the patient was in great distress, and the dressings were removed. M. Boyer then directed him to cough; dark blood flowed copiously from the wound. The left side of the thorax was observed to be more prominent than the other, and the intercostal spaces were obliterated: a portion of air entered the chest in lieu of the blood evacuated. The dressings were again applied. In the evening, the oppression continuing, twenty leeches were applied to the anus.

On the 12th and 13th, he continued with gradually increasing oppression of respiration, anxiety, and general distress.

On the 14th, M. Boyer introduced a probe through the wound into the chest; immediately the blood sprung to the height of several feet. He then removed the instrument, and tried to introduce his little finger. This brought on a kind of spasmodic action of the respiratory muscles, and the blood escaped as before. Air entered to occupy the place of the blood; the pulse became extremely feeble, and little or no relief was given to the breathing. About three o'clock in the morning he died.

*Examination.*—An incision was made at the inferior and back part of the thorax, through which about a pound and a half of blood was extracted. The chest was then opened with great precaution. An aperture was found in the pericardium, which was thickened and inflamed throughout its whole extent: it adhered to the heart only at the edges of the wound. It contained pus, particularly at the lower part, where the quantity was considerable: it was thick and greenish. An opening, rather less than that in the pericardium, existed in the left ventricle: this ventricle was removed by two incisions parallel to the septum. A stilette, introduced from without inwards, penetrated into its interior, and pushed out a little clot of blood. The inner aperture was extremely small. A false membrane, which lined the surface of the pericardium and the heart, extended itself across the wound; the heart appeared to be becoming inflamed, being thickened and very hard. There was no blood in the pericardium; no artery that could be seen was wounded.—*Ibid.*

